# Carboxylate-Modified Magnetic Bead (CMMB)-Based Isopropanol Gradient Peptide Fractionation (CIF) Enables Rapid and Robust Off-Line Peptide Mixture Fractionation in Bottom-Up Proteomics

**DOI:** 10.1074/mcp.RA120.002411

**Published:** 2021-01-19

**Authors:** Weixian Deng, Jihui Sha, Kathrin Plath, James A. Wohlschlegel

**Affiliations:** 1 David Geffen School of Medicine, Department of Biological Chemistry, University of California Los Angeles, Los Angeles, California, USA; 2 Molecular Biology Interdepartmental Graduate Program, University of California Los Angeles, Los Angeles, California, USA

**Keywords:** Peptide fractionation, SP3, proteomics, AP, affinity purification, CIF, CMMB-based isopropanol gradient peptide fractionation, CMMB, carboxylate-modified magnetic bead, PSM, peptide spectral match, RP, reverse phase, SA, streptavidin, SCX, strong cation exchange

## Abstract

Deep proteome coverage in bottom-up proteomics requires peptide-level fractionation to simplify the complex peptide mixture before analysis by tandem mass spectrometry. By decreasing the number of coeluting precursor peptide ions, fractionation effectively reduces the complexity of the sample leading to higher sample coverage and reduced bias toward high-abundance precursors that are preferentially identified in data-dependent acquisition strategies. To achieve this goal, we report a bead-based off-line peptide fractionation method termed CIF or carboxylate-modified magnetic bead–based isopropanol gradient peptide fractionation. CIF is an extension of the SP3 (single-pot solid phase–enhanced sample preparation) strategy and provides an effective but complementary approach to other commonly used fractionation methods including strong cation exchange and reversed phase–based chromatography. We demonstrate that CIF is an effective offline separation strategy capable of increasing the depth of peptide analyte coverage both when used alone or as a second dimension of peptide fractionation in conjunction with high pH reversed phase. These features make it ideally suited for a wide range of proteomic applications including the affinity purification of low-abundance bait proteins.

## Highlights

• CIF provides a new on-bead offline peptide fractionation method based on HILIC.
• CIF offers a complementary dimension to RP for peptide fractionation.
• CIF can be seamlessly integrated with SP3 protein sample desalting.
• A machine learning model is able to predict CIF peptide fractionation patterns based on its peptide sequence.

## Graphical Abstract

**Figure:**
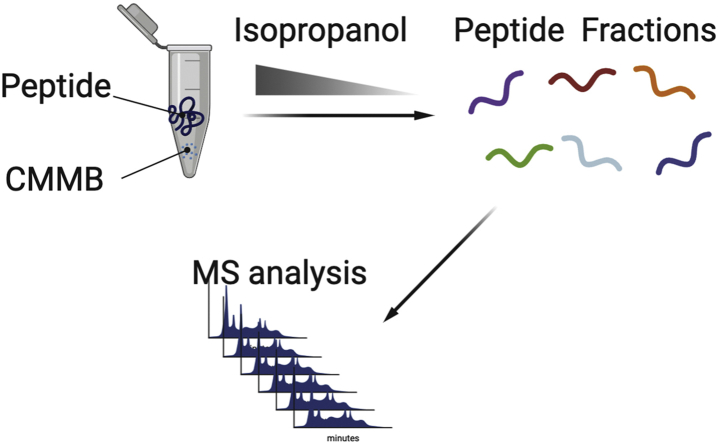

## In Brief

We propose a novel method for fractionating tryptic peptide mixtures on carboxylate-coated magnetic beads. It is an extension of the previously reported SP3 (single-pot solid phase–enhanced sample preparation) protein and peptide cleanup method and provides an effective but complementary approach to other commonly used fractionation methods including strong cation exchange (SCX) and reversed phase (RP)-based chromatography.

Shotgun mass spectrometry has been the major strategy for bottom-up proteomics for decades (1). This technique involves analyzing a population of proteolytically digested peptides that are eluted from the reversed-phase (RP) separation into the mass spectrometer and then selecting the most intense ones for fragmentation to generate sequence information. Owing to limitations in scan speed, however, mass spectrometers are unable to fragment and scan all of the precursors eluting at a given time into the mass spectrometer, resulting in undersampling of low-abundance peptides. This problem becomes more severe as the peptide mixture becomes more complex and ultimately results in reduced proteomic depth for the analysis of many complex biological samples. In addition, the large number of coeluting peptide precursors also leads to ion suppression, which further limits the ability to identify and quantify low-abundance precursors (2). These issues are typically addressed, at least in part, by using chromatographic methods to reduce the complexity of the mixture to enable the mass spectrometer to isolate and fragment the majority of peptides coeluting at any given retention time. Strong cation exchange (SCX) and high pH RP chromatography have emerged as the two most common strategies for reducing peptide complexity offline before analysis by LC-MS/MS (2, 3). In addition, owing to lack of accessibility to HPLC equipment, many laboratories use spin-column, stage-tip, or solid-phase extraction cartridges filled with matrix material (C18 for RP, benzenesulfonic acid bonded sorbent for SCX) instead of an HPLC, in essence, sacrificing some fractionation efficiency for speed and ease of use.

Although SCX fractionation methods have strong orthogonality to low pH RP chromatography (4), the use of salt in the mobile phase for elution requires an extra desalting procedure to make it compatible with LC-MS. Moreover, SCX chromatography often suffers from inefficient peptide recovery because of secondary interactions with the SCX sorbents that reduce the recovery of hydrophobic peptides (5). Compared with SCX, high pH RP fractionation requires no additional cleanup steps for fractionated products that limits sample loss. Like SCX, however, high pH RP suffers from incomplete peptide recovery with this material loss becoming more evident in samples with low amounts of peptide (6). Considering both offline high-pH RP and online low-pH RP utilize a similar peptide binding matrix and buffers (besides pH), the orthogonality of fractions for low-pH RP is not ideal (7).

To circumvent the disadvantages of SCX and RP chromatography, a rapid, robust fractionation method that is compatible with low sample amounts and orthogonal to online low-pH RP chromatography is needed. In a previous study, it was shown that proteins and tryptic peptides can be immobilized on the hydrophilic surface of carboxylate-modified magnetic beads (CMMBs and also widely known as SP3) in an unbiased manner using a high concentration of the organic solvent (8, 9, 10). This method is derived from a mechanism similar to hydrophilic interaction liquid chromatography (11) or electrostatic repulsion hydrophilic interaction chromatography (12). It features high material recovery and high binding capacity and can be easily integrated into a variety of proteomics applications. Using the CMMB/SP3 technology, peptides are eluted from the beads when the acetonitrile (ACN) concentration is decreased below 90% with no detectable retention. However, owing to the narrow ACN concentration window over which peptide elution occurred, only limited success was reported when peptide fractionation was attempted using CMMB/SP3 (8). In this study, we describe a novel CMMB-based isopropanol gradient peptide fractionation method that we termed CIF that allows the elution of peptides into fractions using a step-wise isopropanol gradient. This strategy not only leverages the high binding capacity and low material loss advantages of CMMB but also achieves effective offline peptide-level fractionation, thus facilitating deeper proteomic coverage and improved analysis of high dynamic range samples.

## Experimental Procedures

### Cell Culture and Tryptic Peptide Preparation

HEK293 cells were cultured in high glucose and glycine DMEM containing 10% FBS and 1% penicillin-streptomycin and then trypsinization for harvesting. Incubating cells in the lysis buffer (8 M urea, 0.1 M Tris HCl, pH 8.0) at 4 °C for 30 min followed by centrifugation to clarify the sample. Two milligrams of protein were reduced and alkylated by sequentially incubating with 5-m ris(2-carboxyethyl)phosphine and 10-mM iodoacetamide or 30 min at room temperature (RT) in the dark. The protein sample was then diluted fourfold with 0.1 M Tris HCl, pH 8.0, to reduce the final urea concentration to 2 M before incubating overnight with at 37 °C with trypsin protease at ratio of 1:100. SP3 was reported not suitable for cleanup of high quantity of proteins (high microgram or milligram quantities) (9). So, peptide digests were desalted using Pierce C18 tips (100-μl bed volume, cat. 87784), dried, and then reconstituted in water.

### Peptide Recovery Assay

For each elution concentration tested, 1.7 μg of peptides were reconstituted in 10 μl of water, mixed with 5 μl of CMMB (GE Healthcare: 65152105050250, GE Healthcare: 45152105050250, mixed at 1-to-1 ratio) followed by 300 μl of ACN, which raises the final ACN concentration to 95% and allows peptide binding to CMMB. Peptides were eluted from CMMB by incubation with 30 μl of the elution buffer containing varying amounts of isopropanol (95%, 90%, 85%, 80% 75%, 70%, 0% of isopropanol) in a thermomixer for 15 min. Peptide concentrations were determined for each sample by measuring their absorbance at 205 nm on a NanoDrop 3000 spectrophotometer.

### Optimization of Bead Amount for CIF

To optimize the ratio of beads required to fractionate 20 μg of peptides using CIF, we tested the ability of 1 μl, 5 μl, 20 μl, and 50 μl of 50 μg/μl CMMB to fractionate 20 μg of peptides. Twenty microliter-beads (∼1000 μg) showed the greatest number of identified peptides (supplemental Fig. 1, *A* and *B*), whereas 5 μl beads (∼250 μg) showed only marginally fewer identified peptides. We conclude that CIF is effective over a broad range of peptide-to-bead mass ratios ranging from 1:12.5 to 1:50.

### CIF

**Fig. 1:**
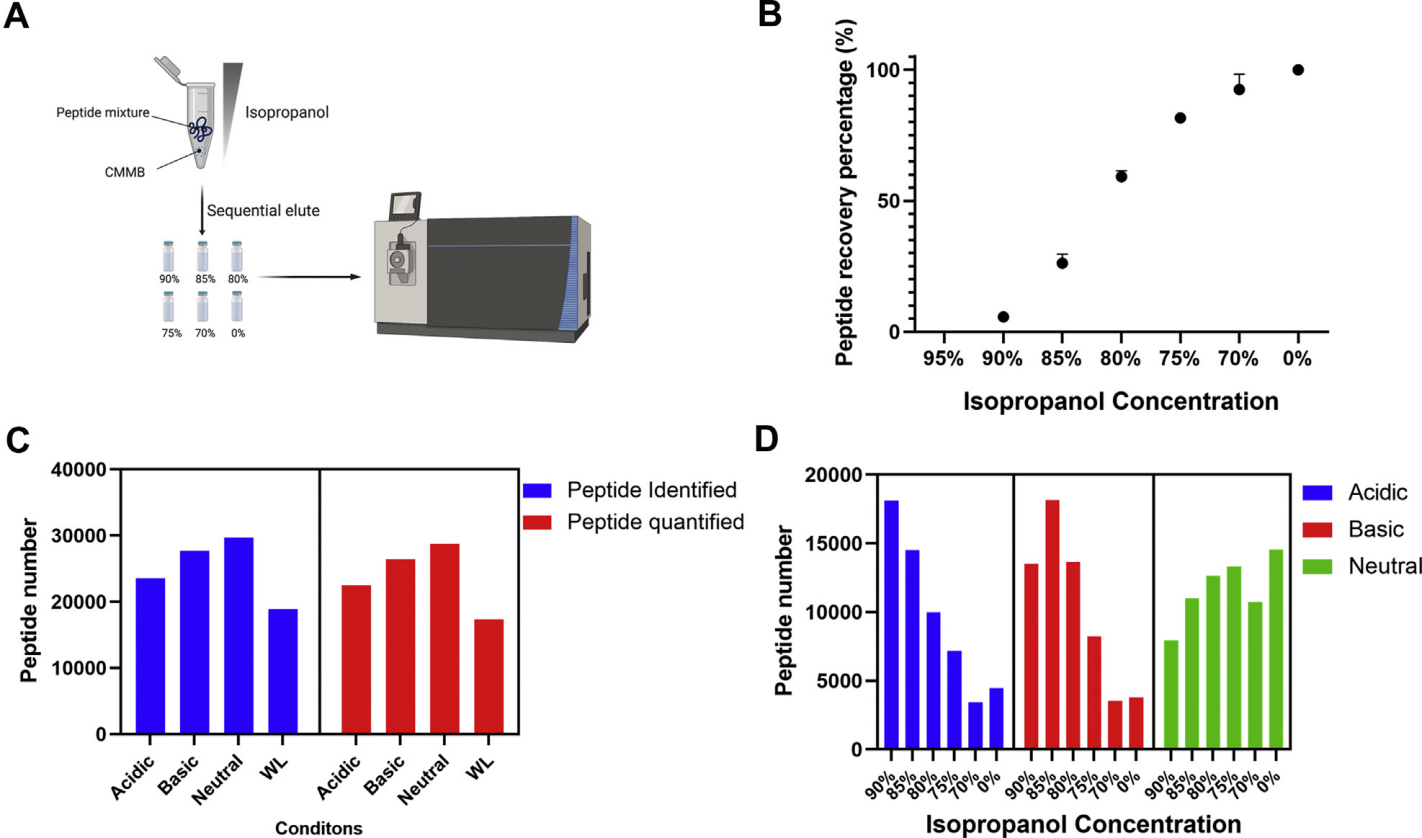
**Effective stepwise elution of peptides from CMMB using isopropanol at neutral pH**. *A*, the schematic of CIF workflow. *B*, fraction of peptides eluted from CMMB at different isopropanol concentrations, 95% (mean: 0, SD: 0.39), 90% (mean: 5.74, SD: 0.74), 85% (mean: 26.11, SD: 3.35), 80% (mean: 59.13, SD: 2.32), 75% (mean: 81.61, SD: 1.05), 70% (mean: 92.32, SD: 5.83), 0% (mean: 99.88, SD: 0.64), (n = 3). *C*, the number of unique peptides identified or the number of peptides quantified after LC-MS/MS analysis of peptides fractionated by CIF in acidic (isopropanol solution with 5% formic acid), neutral (isopropanol solution with water) or basic (isopropanol solution with 50-mM triethylamine bicarbonate) pH conditions. WL is the unfractionated control. (n = 1). *D*, the number of unique peptides identified by LC-MS/MS analysis in each isopropanol fraction after fractionation by CIF under acidic, basic, and neutral pH conditions (n = 1). CIF, carboxylate-modified magnetic bead–based isopropanol gradient peptide fractionation; CMMB, carboxylate-modified magnetic beads; WL, whole lysate.

For each experiment, a defined amount of digested peptide (7 μg for the pH comparisons, 100 μg for comparisons between fractionation methods, and 20 μg for modeling the CIF elution concentration experiment) was bound to CMMB by incubating the peptide/CMMB mixture in 95% ACN for 10 min at RT. Peptides were eluted by sequentially incubating the CMMB with 30 μl of each elution buffer (90%, 85%, 80% 75%, 70%, 0% of isopropanol) and pipetting up and down 15 times (pipetting up and down can be replaced by shaking on a shaker at 1200 rpm for 10 min). Each elution step was repeated once to ensure no residual peptides were carried over into the next elution. Eluted peptides were dried by vacuum centrifugation and reconstituted in 5% formic acid for LC-MS/MS analysis (Fig. 1*A*). To test different pH conditions, 50-mM triethylamine bicarbonate was added to the isopropanol elution to create high-pH conditions, whereas 5% formic acid was added to the isopropanol elution to generate low-pH conditions.

### High-pH RP Fractionation

High-pH RP fractionation was performed according to the manufacturer's instructions (Pierce High-pH Reversed-Phase Peptide Fractionation Kit, catalog number: 84868). Essentially, 100-μg peptides were bound to the resin in the spin column and then eluted by stepwise incubations with 300 μl of increased ACN concentrations. Fractions were then dried by vacuum centrifugation and reconstituted in 5% formic acid for mass spectrometry analysis.

### RP-CIF 2D Fractionation

For eight-fraction RP-CIF 2D fractionation, 100 μg of the peptide mixture was bound to the resin in the spin column and then eluted stepwise with 300 μl of increasing ACN concentrations. Subsequently, eluate fractions 1 and 5, 2 and 6, 3 and 7, and 4 and 8 were combined pairwise into four total fractions, dried by vacuum centrifugation, and reconstituted in 30 μl of water. Each combined RP fraction was bound to 10-μl CMMB and eluted in two steps (80% isopropanol and water) as described in the CIF section. For 16-fraction RP-CIF 2D fractionation, conditions were identical except RP fractions were not combined before applying CIF.

### APEX2-Based Proximity Labeling

The APEX2-Oct4 fusion protein coding sequence was cloned into the pMX retro-viral packaging vector and then transiently transfected into HEK293 cells with Lipofectamine 3000. Five hundred micro molar biotin-phenol was added to the media 18 h after transfection and incubated at 37 °C for 30 min. Then peroxidase reaction was activated by adding H_2_O_2_ to 1 mM and incubating at RT for 1 min. The reaction was quenched by washing cells three times with quencher containing PBS (10-mM sodium azide, 5-mM Trolox, 10-mM sodium ascorbate). Cells were harvested by trypsinization and then flash-frozen in liquid nitrogen.

### Streptavidin Pull-Down

Cells are lysed in RIPA buffer (50-mM Tris HCl, pH 7.5, 150-mM NaCl, 0.1% SDS, 0.5% sodium deoxycholate, 1% Triton X-100) supplemented with protease inhibitor cocktail (Roche) and Benzonase (1 μl of 250 U/μl) and incubated at 37 °C for 20 min. Lysates were clarified by centrifugation, quantitated using the Pierce 660-nm protein assay, and 1 mg of protein was incubated with 300 μl of high-capacity streptavidin (SA) beads (Thermo Fisher) for each sample at RT for 1 h. SA beads were then washed three times with RIPA buffer, once with 1 M KCl, once with 2 M urea in 25-mM Tris HCl, pH 8.0, and three more times with RIPA buffer. Bound proteins were then reduced, alkylated, and digested on beads with Lys-C and trypsin. The supernatant from the on-bead digestion was then transferred to another tube, bound to SP3/CMMB beads by the addition of ACN to a concentration of 95%, and either eluted from CMMB in water or fractionated using the CIF protocol into three fractions (85%, 75%, and 0% isopropanol elution steps). Although it is difficult to measure the protein abundance on beads in this type of analysis, we estimate that there is less than 1 μg of protein (not including SA) based on other experiments using comparable purification strategies.

### LC-MS Data Acquisition

A 75-μm × 25-cm homemade C18 column was connected to a nano-flow Dionex Ultimate 3000 UHPLC system. The 70-min gradient of increasing ACN was delivered at a 200 nl/min flow rate as follows: 1% ACN phase from minutes 0 to 6, 6 to 25% ACN from minutes 6 to 55, 25 to 32% ACN from minutes 55 to 63.5, 32 to 80% ACN from minutes 63.5 to 67, and then 1% ACN from minutes 68 to 70. An Orbitrap Fusion Lumos Tri-brid mass spectrometer was used for data acquisition. Full MS scans were acquired at 120 K resolution with the automatic gain control target set to 2e5 and a maximum injection time set to 100 ms. MS/MS scans were collected at 15K resolution after isolating precursors with an isolation window of 1.6 m/z and HCD-based fragmentation using 35% collision energy. For data-dependent acquisition, a 3-s cycle time was used to acquire MS/MS spectra corresponding to peptide targets from the preceding full MS scan. Dynamic exclusion was set to 25 s.

### Database Search

MS/MS database searching was performed using MaxQuant (1.6.17.0) against the human reference proteome from EMBL (UP000005640_9606 HUMAN *Homo sapiens*, 20600 entries, released in 2020_04). The search included carbamidomethylation on cysteine as a fixed modification and methionine oxidation and N-terminal acetylation as variable modifications. The digestion mode was set to trypsin and allowed a maximum of two missed cleavages. The precursor mass tolerances were to 20 and 4.5 ppm for the first and second searches, respectively, whereas a 20-ppm mass tolerance was used for fragment ions. Data sets were filtered at 1% false discovery rate at both the peptide spectral match (PSM) and protein level. Peptide quantitation was performed using MaxQuant's LFQ mode.

### Modeling the CIF Elution Profile

We used peptide and elution information from the CIF data set to identify the physicochemical properties of peptides that determine their elution using CMMB. The R package, Peptides (v2.4.1), was used to calculate the aliphatic index (13), peptide charge at given pH, peptide isoelectric point (14), instability index (15), and hydrophobicity (16) as well as 18 parameters related to amino acid composition including the number and mole percentage of nine classes of amino acids. These variables were normalized using the Max-Min normalization method to ensure all the variables values were within (0,1). The model was generated using all three replicates of the CIF data that were combined by assigning the isopropanol elution concentration in which the peptide displayed maximum LFQ intensity across all replicates and concentrations. Peptides eluted in 0% isopropanol were removed because it was not possible to determine a narrow range of isopropanol over which those peptides were eluted. After combining and preprocessing the data, we obtained a matrix with 39,225 peptide sequences or rows, 23 columns of variables, and an observed elution isopropanol concentration. We then separated the data set into two parts by randomly assigning two-thirds of the rows to the training set and one-third to the test set. Using the training set, we trained a Lasso regression with tenfold cross-validation using the R package glmnet (v3.0–2) (17). We selected the model where the lambda value provided the most regularized model such that error was within one standard error of the minimum and then removed features that contributed minimally to the model (Table 1). An R script that generates peptide properties and predicts the isopropanol elution concentration can be found here: https://github.com/weixiandeng/CIFpredictor.

**Table 1:** Peptide fractionation pattern multivariable linear model

Variables	Coefficients
(Intercept)	76.62
Charged No.	−21.05
Charge	11.87
Hydrophobicity	8.30
Polar No.	−6.82
Acidic mole %	−5.34
a-Index	5.27
Tiny mole %	−3.83
Nonpolar mole %	0.85
Instability index	−0.19
RMSE	3.82
R^2^	0.70

Physical chemistry peptide properties (gray) and peptide composition properties (green shaded) contribute to the model.

### Calculation of the Distribution Index

The distribution index is calculated by the following equation for which we partition the 70-min gradient into 70 equal bins and denote the number of PSM counts in each bin as $P_{j}$.

$$Distribution\;index=\frac{\sum_{i=1}^{70}P_{i}}{\sum_{j=1}^{70}P_{j}}\times 100\text{\%}$$

$$P_{j}\;is\;PSM\;count\;with\;in\;each\;retention\;time\;bin,P_{i}=\left\{ \begin{array}{l} P_{i}=P_{j},P_{i}\leq \frac{\sum_{j=1}^{70}P_{j}}{70}\\ P_{i}=\frac{\sum_{j=1}^{70}P_{j}}{70},P_{i}>\frac{\sum_{j=1}^{70}P_{j}}{70} \end{array}\right.$$

### Experimental Design and Statistical Rationale

**Fig. 2:**
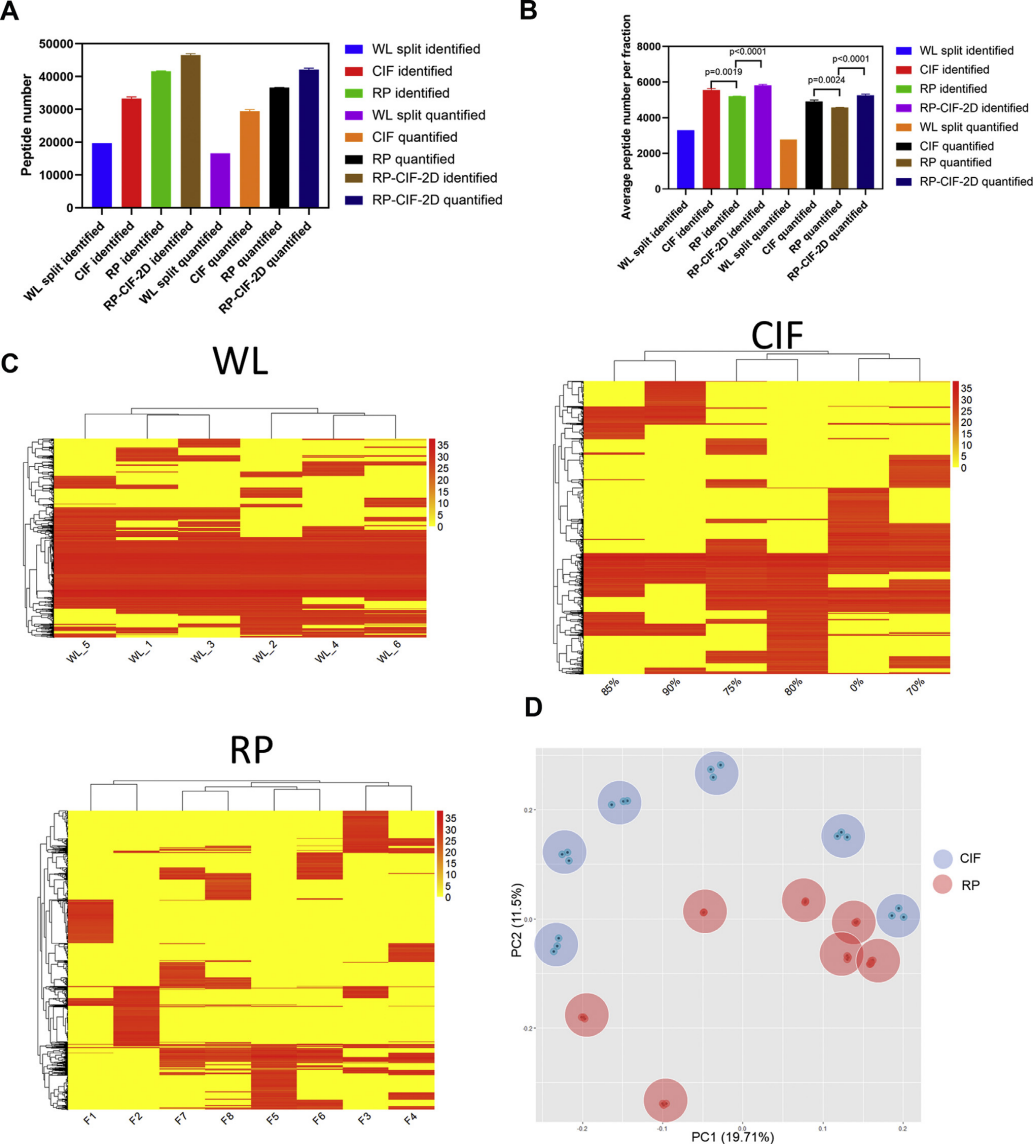
**CIF effectively fractionates complex peptide mixtures leading to improved depth of proteome coverage**. *A*, the number of unique peptides identified or the number of peptides quantified by LC-MS/MS analysis of 100 μg of digested peptides either evenly split into six fractions (WL, no replicate) or fractionated using CIF RP or eight-fraction RP-CIF-2D (n = 3). *B*, identical to (*A*), except the number of identified peptides, the number of quantified peptides is normalized by the number of fractions analyzed. *C*, the heatmaps of quantified peptides in different fractions from WL (replicates of unfractionated sample), CIF (six fractions), and RP (eight fractions) samples. Each heatmap is derived from one representative sample. *D*, principal component analysis (PCA) of peptide intensities from each fraction for both CIF and RP experiments including replicates (n = 3). Each *dot* represents one replicate LC-MS/MS while each shaded *circle* represents all of the replicates corresponding to a specific fraction. CIFs are shaded in *red*, whereas RP fractions are shaded in *blue*. CIF, carboxylate-modified magnetic bead–based isopropanol gradient peptide fractionation; RP, reversed phase; WL, whole lysate.

**Fig. 3:**
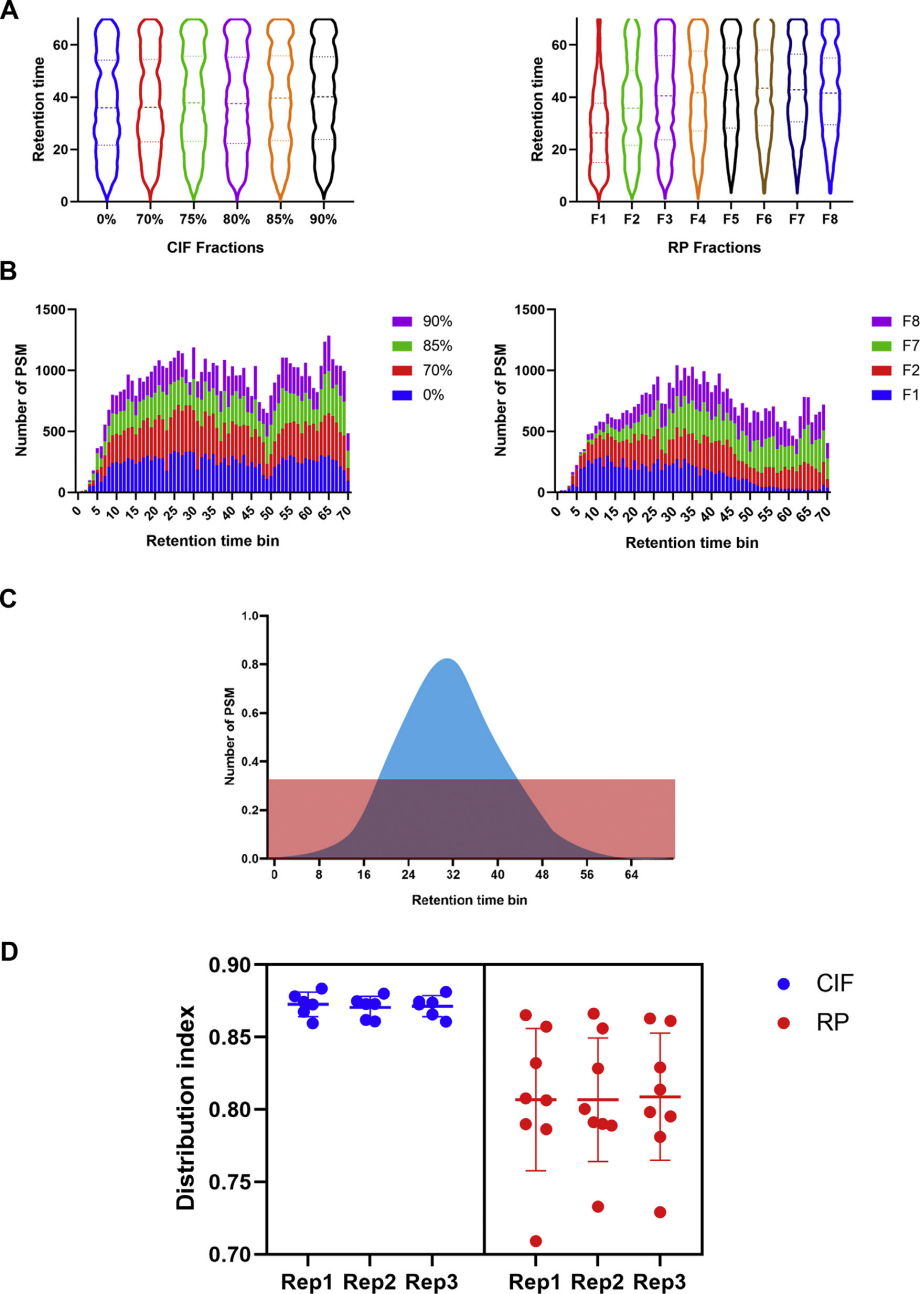
**Peptides from CIFs are more evenly distributed than peptides from basic RP fractions across the LC-MS/MS online RP gradient**. *A*, the violin plot of PSM number distribution at corresponding retention time in each fraction of CIF (*left*) or RP (*right*) fractionation method. *B*, the histogram overlay of identified PSMs at the corresponding retention time divided into 1-min bins for the first two (*blue* and *red*) and last two (*green* and *purple*) fractions of the CIF-based (*left*) or RP-based (*right*) fractionation. These data are derived from one representative replicate out of three total replicates per condition. *C*, the schematic representing the distribution index where *red* indicated the idealized distribution if peptides elute evenly across the entire gradient, *blue* indicating a typical peptide elution distribution, and the area of the overlap representing the distribution index. *D*, the distribution index of replicate fractions from the CIF (*blue*) and RP (*red*) separations (n = 3). CIF, carboxylate-modified magnetic bead–based isopropanol gradient peptide fractionation; RP, reversed phase.

Peptide recovery experiments were performed in three technical replicates. CIF experiments exploring different pH conditions (Fig. 1, *C* and *D*) were single replicate-relative comparisons that were validated in subsequent experiments. All comparisons between CIF, RP, and RP-CIF-2D fractionation experiments (Figs. 2 and 3) were performed using three technical replicates from the same HEK293 digested lysate. Statistical comparison of peptides identified in Figure 2*B* were conducted using an unpaired Student's *t* test. APEX2-based proximity labeling experiment was performed using two technical replicates. Nontransfected but otherwise identically treated cells served as the negative control for the analysis. MSStats (3.10) was used to analyze the MaxQuant LFQ data in the APEX2-Oct4 proximity labeling experiment to statistically assess protein enrichment. Equalized medians were used for normalization and the Tukey median polish method was used for protein summarization. *p*-Values for *t* tests were corrected for multiple hypothesis testing using the Benjamini-Hochberg adjustment. For proteins absent in either condition, the fold change was imputed based on its abundance in the detected condition.

## Results

### Isopropanol-Based Fractionation of Peptides on CMMB

According to Hughes *et al*. (8), the majority of bound peptides are coeluted from CMMB/SP3 when the ACN concentration drops below 87%, providing only a narrow useful range for stepwise elution. To identify other solvents capable of eluting peptides in a graded manner from CMMB, we tested methanol, ethanol, and isopropanol as candidates to replace ACN. For each solvent, we bound tryptic peptide digests from HEK293 whole-cell lysates to CMMB in the presence of 95% ACN (by volume). We then eluted the peptides from the CMMB by incubating with stepwise decreases in the solvent concentration (95%, 90%, 85%, 80%, 75%, 70%, and 0%) (Fig. 1*A*). Peptide recovery for each solvent at each elution condition was measured by peptide absorbance at 205 nm. Methanol, ethanol, and ACN showed similar elution profiles in which nearly all peptides were eluted across a narrow range of solvent (data not shown). In contrast, peptides were strongly bound to the beads in 95% ACN and eluted gradually from the beads by decreasing isopropanol concentrations (Fig. 1*B*). We also examined the ratio of beads to digested protein required for optimal separation using CIF and determined a bead-to-protein ratio of 1:12.5 to 1:50 resulted in optimal fractionation and peptide identification (supplemental Fig. 1, *A* and *B*). These characteristics suggested that isopropanol could be useful as a solvent for offline peptide fractionation on CMMB before LC-MS analysis and that this fractionation worked over a wide bead:protein ratio.

We also examined peptide elution from CMMB under different pH environments because previous work on hydrophilic interaction liquid chromatography (18) found that pH can affect the binding affinity of peptides to a noncharged matrix. We bound HEK293-derived tryptic peptide digests to CMMB and then eluted peptides stepwise into six fractions using decreasing concentrations of isopropanol (90%, 85%, 80%, 75%, 70%, 0%) across a range of pH levels. Fractionated samples were subjected to LC-MS analysis and compared with unfractionated peptide digests as a control. In Figure 1*C*, we compared the number of unique peptides identified and quantified peptides across different pH conditions relative to a single LC-MS/MS run of the unfractionated control (whole lysate). Both identified and quantified peptides are higher in fractionated samples irrespective of pH. For pH comparisons, neutral pH fractionation generated more peptide identifications relative to the acidic and basic fractions (Fig. 1*C*). In addition, neutral pH showed the most even distribution of peptides across the isopropanol steps, whereas both acidic and basic fractionations resulted in the majority of the peptides being eluted in the early elution steps (Fig. 1*D*). These data suggest that the even distribution of peptides across isopropanol fractions at neutral pH relative to low/high pH explains its improved peptide identification rates. Overall, these findings indicate that neutral pH elution using isopropanol effectively fractionates CMMB/SP3 bound peptides across a broad concentration range and led us to explore its utility in proteomic applications.

### Peptide Fractionation by CIF Increases Proteome Coverage

One of the main benefits of peptide fractionation is improved analyte coverage. Figure 1 showed that CIF produced a higher number of identified peptides and quantifiable peptides relative to an unfractionated sample. However, this increase could have been attributed primarily to the length of the analysis because data were collected for six 70-min gradients for the fractionation experiment (six fractions × 70 min per fraction) compared with only a single 70-min analysis of the unfractionated sample. To distinguish whether the increase in analyte coverage resulted from longer data acquisition or reduced complexity due to the fractionation itself, we compared peptide identifications between a sample in which HEK293-derived tryptic peptides were partitioned into six fractions using CIF and analyzed by LC-MS/MS (six fractions × 70 min of LC-MS/MS per fraction) and compared with a sample in which the tryptic peptides were evenly divided into six unfractionated parts that were analyzed by LC-MS/MS (six unfractionated samples × 70 min of LC-MS/MS per sample). For the CIF method, we identified on average 33,311 unique peptides per sample and could quantify 29,452 of them (Fig. 2*A*). For the unfractionated samples, we identified 19,723 unique peptides of which 16,637 were quantifiable (Fig. 2*A*). These data demonstrate CIF collectively produces 69% more identified peptides and 77% more quantifiable peptides from the same amount of input material and analysis time compared with unfractionated samples. For analyses of individual fractions, we identified and quantified on average 3287 and 2773 peptides, respectively, from an unfractionated sample compared with 5815 and 4909 peptides on average, respectively, from a CIF (Fig. 2*B*). We conclude that CIF is an effective method for peptide fractionation and increases peptide coverage by reducing sample complexity.

We next compared the effectiveness of CIF with high pH RP fractionation, a widely used offline peptide fractionation method. Using the Pierce High-pH Reversed-Phase Fractionation Kit, we fractionated our digested peptide sample into eight fractions following the manufacturer's recommendations. LC-MS/MS analysis of these eight fractions resulted in the identification of 41,638 unique peptides and 36,583 quantifiable peptides compared with 33,311 and 29,452 peptides for the six-fraction CIF analysis (Fig. 2*A*). Although high-pH RP outperforms CIF under these conditions (25% and 24% increase in unique peptides identified and quantified, respectively), these increases likely result from the 33% longer analysis time (eight fractions *versus* six fractions). Consistent with this idea, if we normalize the number of identified and quantified peptides per 70-min gradient, then CIF modestly outperforms high-pH RP (5815 *versus* 5204 identified peptides and 4909 peptides *versus* 4573 quantifiable peptides per 70-min gradient). We conclude that CIF and high-pH RP have comparable efficiency in peptide-level offline fractionation experiments.

Next, we compared the peptide elution pattern across the different fractions for unfractionated (whole lysate) *versus* CIF *versus* high-pH RP. As expected, we find that a large number of abundant peptides are reproducibly identified in replicate analyses of unfractionated samples (Fig. 2*C*). However, CIF and high-pH RP fractionation both produced peptides that are predominantly enriched in only one fraction. Notably, this trend is weaker in CIF than in RP with a significant number of peptides also being enriched in two to three fractions, suggesting that the RP separation has better resolution than CIF. Nonetheless, both CIF and RP are effectively fractionating peptides with discrete populations of peptides being eluted at different concentrations of organic solvent. Importantly, both are effective at simplifying complex peptide mixtures to increase the depth of peptide coverage, making them well-suited for standard proteomic workflows.

To assess fraction-to-fraction reproducibility of CIF relative to high-pH RP, we performed the principal component analysis of the two methods. Consistent with the high degree of reproducibility in peptide identifications between replicates seen in Figure 2, *A* and *B*, the principal component analysis plot shown in Figure 2*D* demonstrates that replicate fractions generated using the same concentration of organic solvent clustered well in both methods. We conclude that both methods are highly reproducible. In summary, we demonstrate that the CIF method is able to reproducibly increase peptide coverage when conducting proteomic analyses on complex peptide mixtures and is an effective alternative to high-pH RP yielding comparable results.

### Peptides from CIFs Are Evenly Distributed Across RP Gradients

Distributing peptides across the entire chromatographic gradient is essential for performing MS/MS on as many different precursors as possible and thus maximize the efficiency of data acquisition. To evaluate how peptides from CIFs were distributed across the subsequent online RP gradient, we plotted the number of PSMs across the gradient for each fraction analyzed using 1-min bins. Figure 3*A* displays the PSM distribution across the LC gradient from each of the fractions CIF (left) and RP (right) fractionation experiment. As is shown in Figure 3*A* (left panel), all six fractions have almost identical shape with PSMs evenly distributed on the retention time space. In comparison, the fractions from an offline high-pH RP separation Figure 3*A* (right panel) have a more biased peptide elution pattern relative to CIF. Moreover, the peptide identifications are biased such that the earlier elution steps (F1 and F2) show more identifications in the early portion of the LC RP gradient while late fractions (F7 and F8) identify more peptides near the end of the gradient (Fig. 3*B*). These data demonstrate that CIF displays better orthogonality than high-pH RP to low-pH RP leading to a more even distribution of peptides across each gradient.

To analyze these chromatographic distributions in a more quantitative manner, we defined the distribution index as a metric for assessing the distribution of PSMs across the chromatographic gradient. Ideally, if PSMs are distributed evenly across the gradient, the PSM distribution would be flat and could be represented as a rectangle in which every bin has the same height (Fig. 3*C*, orange area). In practice, the number of identified PSMs can vary significantly across the chromatographic separation (Fig. 3*C*, blue area) and we can calculate a distribution index (see Experimental Procedures) that measures the extent to which the actual PSM distribution falls into the ideal distribution (Fig. 3*C*, overlapped area).

In Figure 3*D*, we plot the distribution index for each fraction from three replicate CIF or RP-based separations. This plot supports what we observed visually in Figure 3*A*. All six CIFs have a similar distribution index, whereas the distribution index for RP fractions was more variable—consistent with the idea that there is not perfect orthogonality between high-pH and low-pH RP chromatography. Figure 3*D* also shows a higher distribution index for CIFs relative to RP fractions, highlighting the even distribution of peptides across the RP gradient. Together, these data indicate that PSMs in CIFs are more evenly distributed on the ACN gradient axis of LC than RP fractions, which may promote better LC-MS acquisition efficiency.

### CIF Serves as the Second Dimension to RP to Further Increase Sequence Coverage in the Same Analysis Time

Based on the orthogonality between RP and CIF, we posited CIF could be used in a multidimensional fractionation strategy together with RP to further improve proteome coverage. To examine this possibility, we took HEK293 digested lysates fractionated using Pierce high-pH RP spin columns, combined them from eight into four fractions as described above, and then used CIF to fractionate each into two additional fractions using 80% isopropanol and water (eight fractions total). These fractions were analyzed by LC-MS/MS, and the peptide identifications were compared with peptide identifications obtained from eight high-pH RP fractions from the high-pH spin columns before they were combined. As demonstrated in Figure 2*A*, the RP-CIF-2D fractionation method identified and quantified on average 46,519 and 42,092 unique peptides, respectively, corresponding to 11.7% more identified peptides and 15% more quantified peptides compared with RP alone using the same amount of analysis time (eight × 70 min). We also analyzed a similar multidimensional fractionation experiment in which all high-pH fractions were subsequently separated using CIF into two additional fractions generating 16 fractions total. As shown in supplemental Figure 2*A*, the 16-fraction RP-CIF-2D fractionation identified and quantified 46.4% and 54% more peptides, respectively, than RP alone, further highlighting the improved depth of this approach.

### CIF Improves Coverage Depth in AP-MS Workflows

**Fig. 4:**
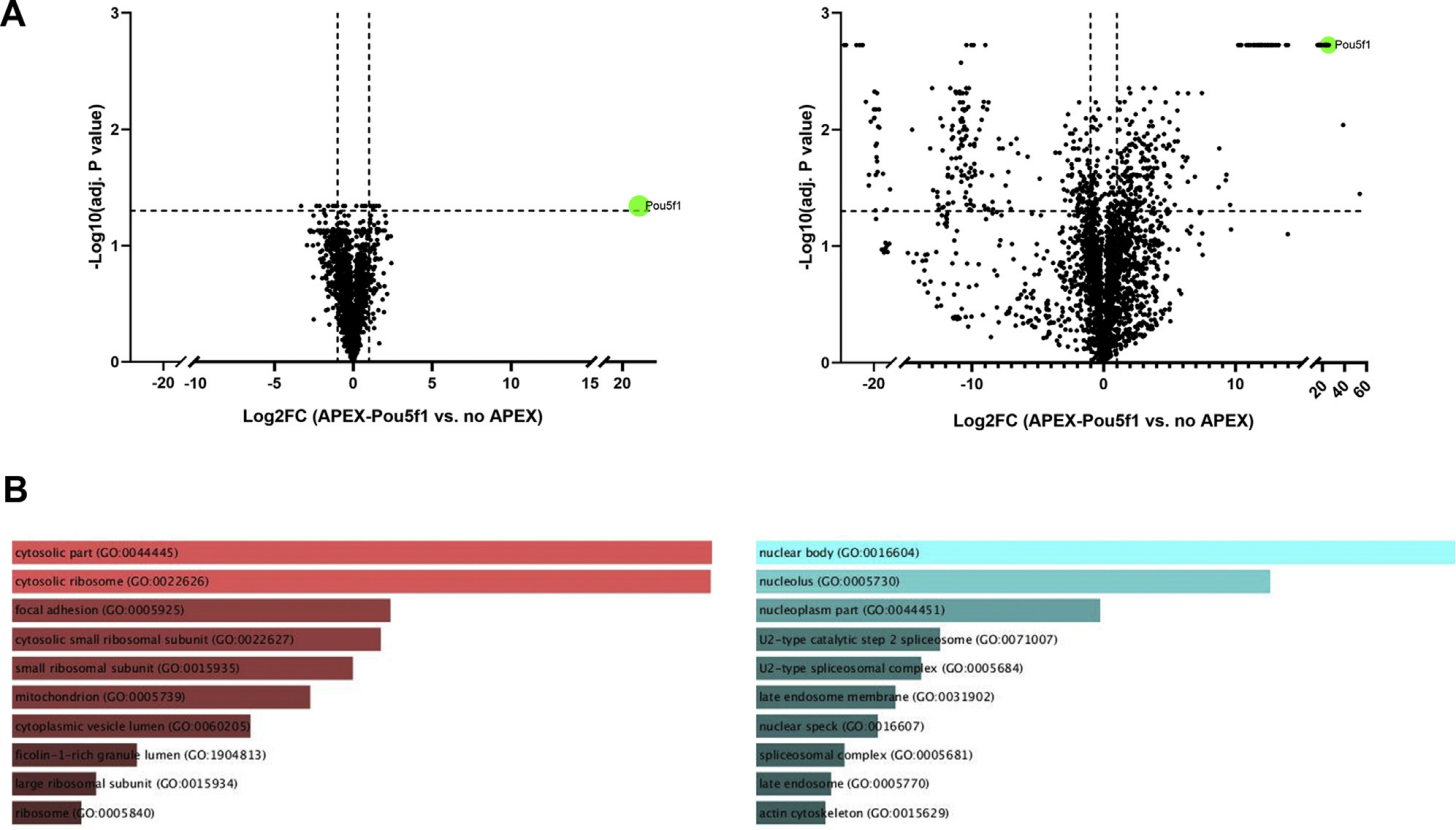
**CIF improves proteome coverage of AP-MS samples.***A*, the volcano plot of proteins enriched in APEX2-Oct4 (Pouf51) proximity labeling experiments. APEX labeling was performed in HEK293 cells expressing APEX2-Oct4 followed by streptavidin purification and LC-MS/MS analysis. Samples were analyzed without prefractionation (*left*) or with CIF (*right*). *B*, Gene ontology analysis (cellular component) of proteins identified as negatively enriched (*left*) or positively enriched (*right*) in APEX-Oct4 experiments CIF (*right*) samples. The samples in (*A*) and (*B*) are from two biological replicates. AP, affinity purification; CIF, carboxylate-modified magnetic bead–based isopropanol gradient peptide fractionation.

Having demonstrated that CIF is a reliable method for peptide-level fractionation in standard proteomics workflows, we next explored applications that might specifically benefit from it. We and others have reported low sample loss as a major strength of CMMB peptide cleanup, leading us to examine whether CIF was suitable for fractionating low-abundance affinity-purified samples to increase depth and data quality. Here, we test CIF's utility in this regard using an APEX proximity labeling experiment. APEX is an engineered ascorbate peroxidase that can label nearby proteins with biotin in the presence of peroxide (19). Leveraging its proximity labeling capacity, researchers fuse it to their protein of interest or to specific localization signal for a cellular compartment to capture protein interactomes or compartment-specific proteomes, respectively. Although the high binding affinity between biotin and SA enables robust capture of labeled proteins and stringent washing conditions during the purification, the SA–biotin interaction creates technical challenges due to (1) the presence of endogenously biotinylated proteins that are captured by the immobilized SA and (2) the high affinity of the interaction often requires denaturation or tryptic digestion to efficiently elute captured proteins, which introduces highly abundant SA into the sample for LC-MS analysis. Both endogenously biotinylated proteins and SA can significantly suppress signal from the less-abundant but physiologically relevant proteins in the sample making efficient fractionation a potential solution for improving the effectiveness of these approaches. However, owing to the low yield that is typical of affinity purification (AP) experiments, commonly used fractionation methods are not generally applied to AP-MS samples. To examine the utility of CIF for APEX-based AP experiments, we examined the proximal interaction of Oct4, one of the Yamanaka transcription factors involved in somatic cell reprogramming (20). Oct4 is a transcription factor that localizes strictly in the nucleus. We expressed APEX2-Oct4 in HEK293 cells and performed proximity labeling while using the parental HEK293 cells as negative control. After labeling, we performed SA AP, on-bead tryptic digestion and then bound the digested peptides to CMMB where peptides were sequentially eluted using three different isopropanol concentrations (85%, 75%, and 0%). In parallel, we conducted a control experiment using CMMB to desalt the samples but without fractionation. Both samples were analyzed by LC-MS. Figure 4*A* shows that only six proteins were enriched in APEX2-Oct4 samples over the no APEX2 control (nontransfected HEK293 cells) (adjusted *p*-value ≤ 0.05, Log2 fold change ≥ 1) for the nonfractionated sample. Of these six proteins, one of them was Oct4 itself and four are localized in the nucleus, making them putative Oct4-proximal proteins. In the fractionated samples, 446 proteins were enriched in APEX2-Oct4 over the no APEX2 control that included Oct4 and 263 nuclear proteins (Fig. 4*B*). These results demonstrate that CIF is effective at fractionating low peptide amounts leading to major increases in sensitivity in proximity labeling experiments.

### CIF Peptide Elution Properties Can Be Predicted by a Multi-Variable Linear Model

**Fig. 5:**
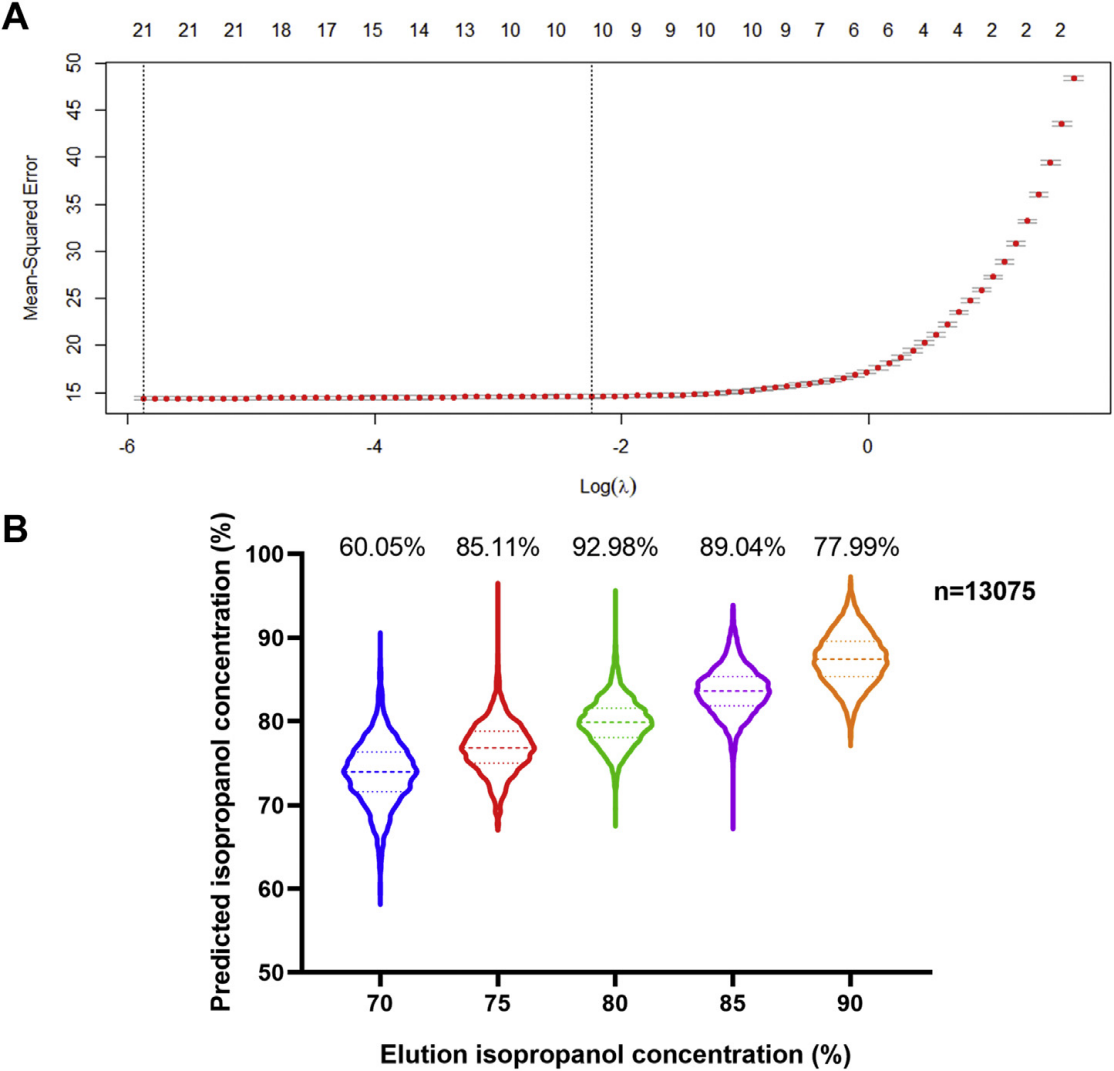
**Peptide elution by isopropanol from CMMB is determined primarily by nine physicochemical properties of the peptides.***A*, the Lasso regression model mean-squared error changes with change of the number of features (*top axis*) and regularization parameter (λ) (*bottom axis*). *B*, the violin plot of the predicted isopropanol concentration necessary to elute peptides from the test set compared with their experimentally determined elution profile from CIF. Test set data points (n = 13,075), the percentage of accurately predicted peptides in each experimentally measured fraction (on the *top* of each column). CIF, carboxylate-modified magnetic bead–based isopropanol gradient peptide fractionation; CMMB, carboxylate-modified magnetic beads.

Figure 2, *C* and *D* show that CIF displays a distinct peptide fractionation pattern from RP. We next explored the determinants of this peptide elution pattern and assessed whether we could predict the peptide elution profile based on peptide sequence alone. To address this question, we attempted to model the elution profile based on peptide sequence. We used the R package Peptides to calculate five physical chemistry properties including aliphatic index, peptide charge at given pH, peptide isoelectric point, instability index, and hydrophobicity as well as 18 amino acid composition parameters that include the number and mole percentage of nine classes of amino acids for a training set of peptides with known elution properties for CIF. We then built a model to describe the relationship between the isopropanol concentration at which a peptide eluted from CMMB and 23 total peptide property variables to assess their relative contribution to the elution profiles (Fig. 5*A*). The model generated has nine variables that contribute significantly to the elution profile (Table 1). The R square for the training set equals 0.70 and the RMSE when cross-validated using a test set of peptides is 3.82, suggesting that the model describes the elution pattern very well. In Figure 5*B*, we plot the predicted isopropanol elution concentration for each peptide in each fraction and considered the prediction correct if the predicted value fell within ± 5% of the observed isopropanol elution concentration. The final prediction accuracies for all fractions ranged from 60.05% to 92.98%, with an average of 81.03%.

Taken together, we built a multivariable linear model that predicts the concentration of isopropanol needed to elute a given peptide based on its sequence and identified the major physiochemical properties that determine this binding. The model indicates that the number of charged amino acids in the peptide sequence, the charge state of the peptide under neutral conditions, and the peptide's hydrophobicity are the key drivers determining which peptides are eluted at what isopropanol concentration.

## Discussion

In this study, we describe a CMMB-based peptide fractionation method that offers several features that provide significant utility in proteomics applications. First, desalting and fractionation are performed in the same tube that minimizes sample loss and facilitates potential automation. Second, the binding capacity of CMMB is high, enabling small beads volumes and hence small elution volumes that also enhances sensitivity and limits losses. Third, CIF is orthogonal to SCX and high-pH RP allowing it to be easily integrated into multidimensional chromatographic schemes. Although we believe that high-pH RP chromatography is likely to remain the superior method for applications for requiring deep proteome coverage, the advantages of CIF outlined here support a strong complementary role for CIF in other common workflows that require high sample throughput or low amounts of starting material (*e.g.*, APs).

One major advantage to CIF is its orthogonality to the acidic online RP separations that are standard in the majority of LC-MS/MS workflows. Considering how evenly peptides are distributed across the LC gradient determines how efficiently data-dependent MS/MS acquisition occurs, the orthogonality of offline separations becomes a determining factor for the effectiveness of the analysis. Based on data in Figure 3, *A* and *C*, we demonstrate that CIF displays excellent orthogonality to RP chromatography in LC-MS/MS applications and is likely the reason for improved peptide identification and quantitation in CIFs. In addition, we take advantage of this orthogonality by demonstrating that CIF and high-pH RP can be used in 2D fractionation experiments to sequentially fractionate peptides offline before LC-MS/MS analysis to further increase peptide coverage.

Another major advantage of CIF is its scalability in terms of peptide input. Standard spin column-based high-pH RP kits typically used for offline fractionation separate input peptides ranging in amount from 10 to 100 μg. However, in the two applications we reported here, CIF is compatible with the fractionation of low-input affinity-purified samples. Specifically, for low-input samples that are particularly sensitive to material loss during processing and which limits fractionation options, we demonstrate CIF retains the ability to efficiency fractionate samples and can improve data quality at those peptide concentration regimes. Based on our experience, fractionation of affinity-purified samples at the level of either cell compartment or peptide significantly improves acquisition of reproducible and biologically meaningful data (data not shown here).

Finally, we built a linear model that predicts the elution properties of a peptide based on its sequence. This model not only sheds light on the mechanism of underlying CMMB peptide-protein binding but also provides a tool for enriching peptides with particular properties. Because the current model assigns very high weights to the number of charged amino acid residuals and peptides that are charged under pH 7, we speculate that CIF might have utility for fractionation of phosphopeptides.

## Data availability

The raw proteomics data are deposited in the MassIVE data repository (https://massive.ucsd.edu) under the identifier MSV000085458. All peptide identification and quantitation data are included in the supporting information.

## Conflict of interest

The authors declare no competing interests.
