# Supplementary material for: Carboxylate-Modified Magnetic Bead (CMMB)-Based Isopropanol Gradient Peptide Fractionation (CIF) Enables Rapid and Robust Off-Line Peptide Mixture Fractionation in Bottom-Up Proteomics
Source: Mol Cell Proteomics. 2021 Jan 19;20:100039. doi: 10.1074/mcp.RA120.002411 (PMC7950212; doi:10.1074/mcp.RA120.002411)
Supplement: Supplemental Figures S1 and S2 [file mmc2.pdf]

Supplemental Figure 1

1A

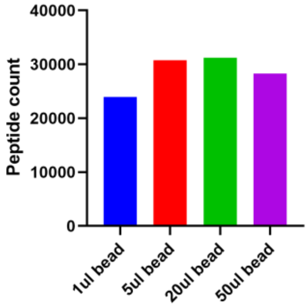

1B

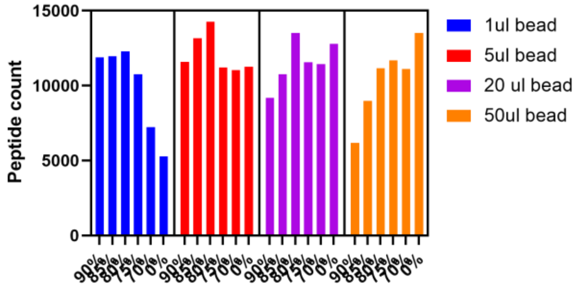

Supplemental Figure 1: (A) Total number of identified peptides in 20 µg CIF fractionated samples using 1 µl, 5 µl, 20 µl and 50 µl CMMB (n=1). **(B)** Number of peptides identified by each fraction of each bead amount CIF experiment.

Supplemental Figure 2

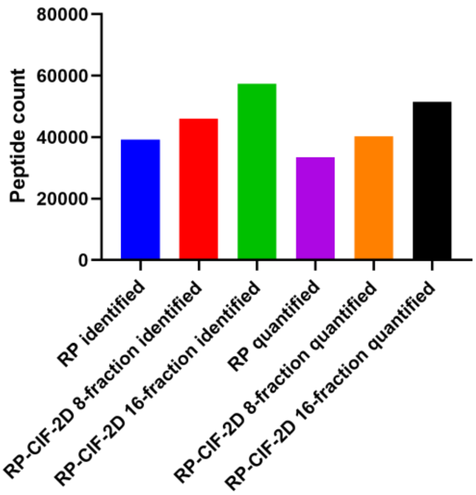

Supplemental Figure 2: Total number of identified peptides fractionated by RP, 8-fraction RP-CIF-2D, and 16-fraction RP-CIF-2D experiments (n=1).
